# Emergency Total Thyroidectomy for Giant Papillary Thyroid Carcinoma Presenting With Acute Airway Obstruction: Management in the Setting of Thyrotoxicosis

**DOI:** 10.7759/cureus.106052

**Published:** 2026-03-29

**Authors:** Kouassi Henry Noel Ahue, Konan Jean N'Dri, Kouide Marius Goho, Kunka Jocelyne Kpan, N’Golo Adama Coulibaly

**Affiliations:** 1 Department of Surgery and Specialty, Faculty of Medicine, Felix Houphouet Boigny University, Abidjan, CIV; 2 Digestive and Endocrinology Surgery Unit, Treichville University Hospital, Abidjan, CIV

**Keywords:** goiter, papillary thyroid carcinoma, respiratory failure, thyroidectomy, tracheomalacia

## Abstract

Giant goiters can lead to tracheal compression with acute respiratory failure. We present a case with a giant goiter as well as acute respiratory failure, which required urgent surgery.

We report the case of a 36-year-old female patient who presented to the emergency room with a giant goiter, which had been present for 12 years and had become compressive for one month, as well as laryngeal dyspnea. For this compressive goiter, she benefited from an emergency thyroidectomy. After histological analysis of the specimen, it was found to be cancerous.

Giant goiters can cause compression of the critical structures of the neck. In cases of compressive goiter, dyspnea is the most common sign of compression and can constitute a life-threatening emergency requiring emergency thyroidectomy. Multi-nodular goiter and thyroid cancers are the two main etiologies found in the literature to be responsible for acute dyspnea due to tracheal compression. Emergency thyroidectomy is the appropriate treatment for asphyxial goiter.

## Introduction

A goiter is defined as a general or localized enlargement of the thyroid gland. According to the WHO, visible forms are estimated to affect approximately 7% of the world's population [1]. Some of these are described as large or giant, surgically defined as weighing more than 100 g [2]. The thyroid gland can be affected by cancer, and according to GLOBOCAN, it is the ninth most common cancer [3]. The most frequent type is papillary carcinoma, which generally presents as a slow-growing disease with a favorable prognosis. However, in rare cases of giant or locally advanced forms, it can cause airway obstruction through tracheal compression, leading to acute respiratory failure, a true emergency that can be life-threatening. Emergency total thyroidectomy then becomes imperative to relieve the tracheal compression. This surgery is performed in a euthyroid state to minimize operative risks. Here, we present the successful emergency management of a patient with giant papillary carcinoma causing acute respiratory failure in the context of thyrotoxicosis [4,5].

## Case presentation

This report describes the case of a 36-year-old female patient who presented to the emergency room with a large goiter occupying the entire anterior surface of the neck, which had been present for 12 years and had become compressive for a month, with laryngeal dyspnea requiring her admission to the emergency room. Furthermore, apart from the respiratory disorder, the patient reported no other signs of compression.

Questioning did not reveal any family history of goiter or irradiation in childhood. A thyroid hormone test performed five days before admission to the emergency department found elevated levels (TSH=0.005 mUI/ml, FT4=49 pmol/L, FT3=22.0 pmol/L) (Table 1).

**Table 1:** Thyroid hormone testing

Laboratory parameters	Unit	Value	Reference	Interpretation
FT4	pmol/L	49	8.6-25	Raised
FT3	pmol/L	22.00	3-9	Raised
TSH	mIU/ml	0.005	0.15-5	Lowered

**Figure 1:**
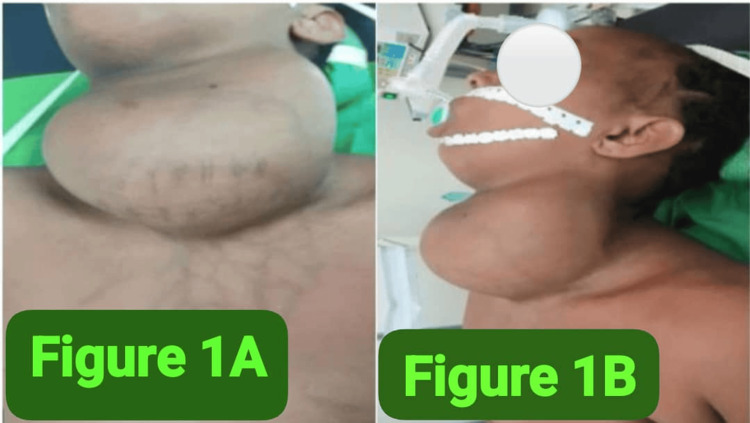
(A and B) Pre-operative image

Clinical examination found a huge goiter occupying the entire anterior region of the neck with collateral venous circulation and cervical lymphadenopathy (Figure 1A-1B). The patient was agitated, displaying respiratory insufficiency (respiratory rate of 39 cycles per minute, oxygen saturation of 85% in ambient air at rest, cyanosis, and laryngeal dyspnea) and signs of clinical dysthyroidism (palpitations, tachycardia at 120 beats per minute, and tremors). Emergency oxygen therapy was initiated, but did not improve the patient's respiratory condition.

Shortly after arriving at the emergency room, the patient showed signs of struggling to breathe, and her consciousness began to deteriorate. During lung auscultation, ventilation in both lungs was reduced. A diagnosis of acute respiratory obstruction secondary to an enlarged goiter was very likely. An alert was triggered, and she was immediately transferred to the intensive care unit, where she was intubated and then taken to the operating room. The team performing the procedure consisted of a senior anesthesiologist, an endocrine surgeon, and an ENT specialist. This clinical presentation posed a dilemma for the team between hyperthyroidism, which contraindicates surgery, and a large, obstructive goiter requiring emergency surgical intervention. After a brief consultation between team members, it was decided to operate on this patient.

Acute respiratory failure was the primary consideration in the decision-making process. It was a challenging intubation due to the severe compression of the airways by the goiter. After two failed tracheal intubations, it was decided to follow plan B from the DAS (Difficult Airway Society) guidelines [6]. This consisted of inserting a supraglottic device (laryngeal mask) to restore ventilation. Then, using a fiberscope, a guided intubation was performed successfully. A standard thyroidectomy was performed. It should be noted that the patient was already taking a synthetic antithyroid drug, neomercazole 20 mg three times a day. To prepare the patient, pre-operative therapy based on injectable propranolol and corticosteroids was instituted before induction. At induction, good analgesia was administered, and curare was given after induction. A 13 cm cervical incision was deemed necessary, which was very hemorrhagic due to cutaneous varices.

**Figure 2:**
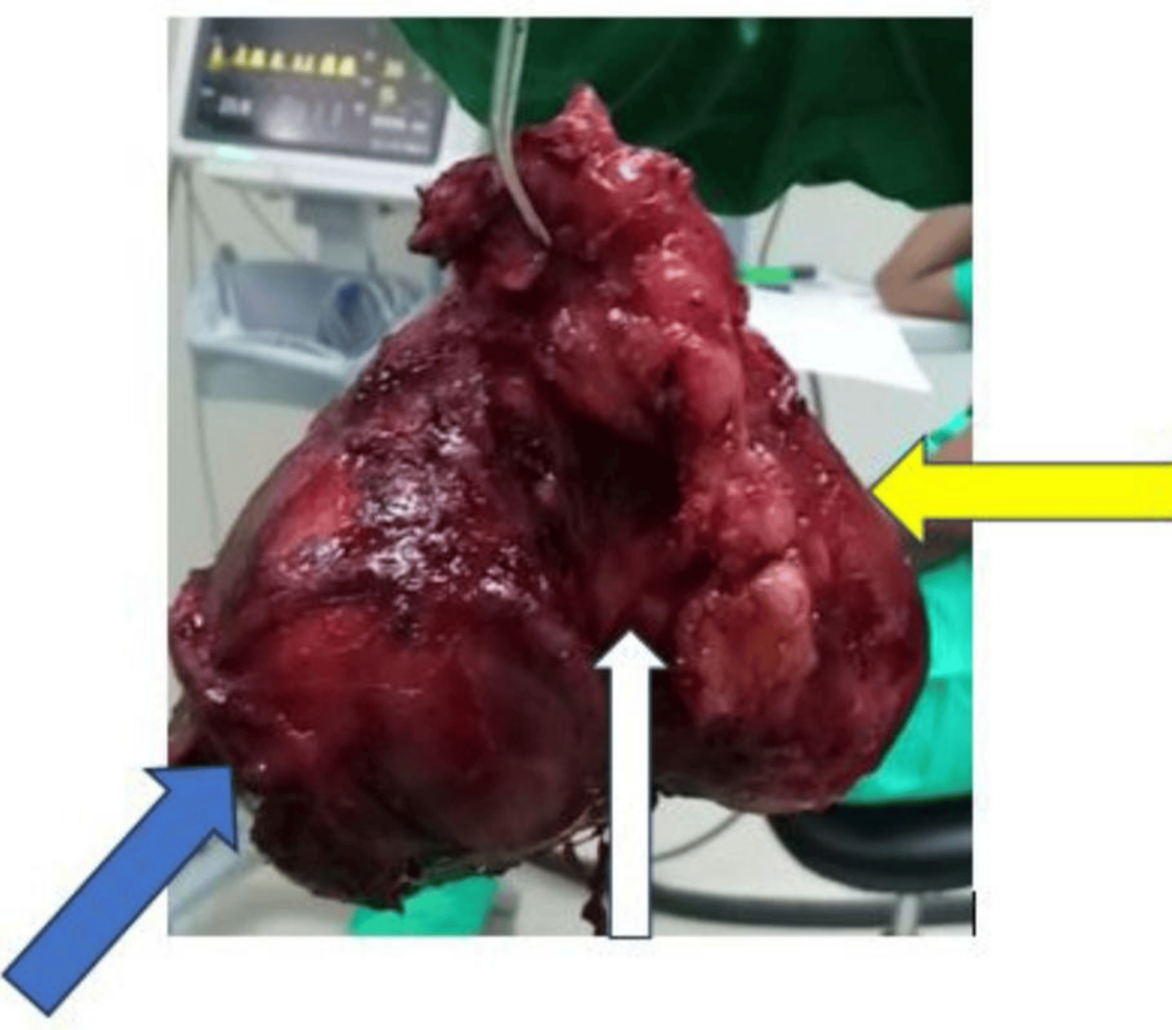
Rear side of the operated specimen Blue arrow: left lobe; yellow arrow: right lobe; white arrow: tracheal insertion area

Exploration of the gland revealed highly lateralized vascular poles. There was no muscular invasion. The goiter measured 14.5 x 10 x 6.5 cm (Figure 2), adhering to and deforming the trachea. Numerous stony lymph nodes of approximately 2 cm were also noted in the jugulocarotid chain. The growth was heart-shaped and multinodular, and very hemorrhagic due to the presence of numerous varices. The gland bled at the slightest touch. Meticulous dissection permitted the identification of the vascular axes, which were then ligated. Only the recurrent laryngeal nerves were visualized due to the size of the goiter and its highly hemorrhagic nature. The parathyroid glands were not visualized. The release of the tracheal mass was difficult, revealing a flaccid and soft trachea. Given this soft trachea, a diagnosis of tracheomalacia was made. The possibility of a tracheotomy was discussed, but we opted for prolonged intubation (48 hours) due to the presence of pre-operative hyperthyroidism. For the team, this was the safest decision. This prolonged intubation was accompanied by high-dose corticosteroid therapy. Extubation was performed 48 hours postoperatively.

**Figure 3:**
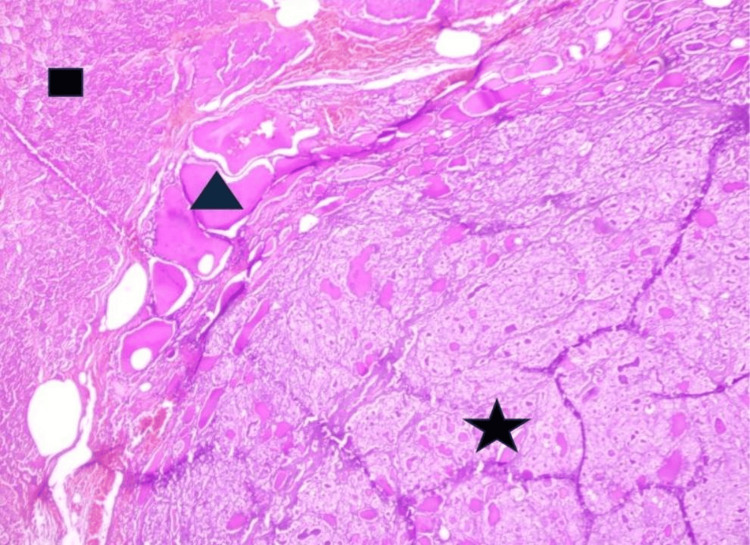
Papillary carcinoma with vesicular architecture (black star) and a normal residual stump of thyroid tissue (black triangle) and muscle tissue (black square)

The post-operative course was uneventful. Post-operative serum calcium levels were normal. Histological analysis of the thyroid showed a papillary carcinoma weighing 245 grams (Figure 3). The patient was discharged on postoperative Day 5 with normal thyroid function and calcium levels. Follow-up at three months, six months, and one year was satisfactory. Standard thyroid replacement therapy with levothyroxine 100 mcg/day was initiated after a delay of 45 days. Monthly thyroid hormone checks were normal for six months.

## Discussion

Goiter is a particularly common pathology that affects approximately 15.8% of the world population [2]. It is generally a familial disease, the frequency of which increases with age, and affects women more frequently than men. Goiter is a term used to describe an enlargement of the thyroid gland of any cause and generally presents with swelling in the anterior neck region with or without compressive symptoms of thyroid dysfunction, or malignant degeneration [7]. A giant goiter is defined as any goiter weighing more than 100 grams [2]. Giant goiters are exposed to compression of the critical structures of the neck. Goiters with compressive symptoms are characterized by appearing in patients older than 55 years with a goiter of more than 10 years’ evolution [8].

In our patient's case, early intervention would not have led to all these problems; this case highlights the importance of screening and ensuring access to care for patients in rural areas. Huge goiters are common in iodine-deficient endemic regions. They are of concern to the surgeons because of the anticipated risk of difficult dissection and increased chances of surgical complications. Similarly, they are of concern to the anesthesiologists because of anticipated intubation-related difficulties and post-thyroidectomy tracheomalacia [9].

In compressive goiter, dyspnea is the most common sign of compression [10] and can constitute a real life-threatening emergency requiring emergency thyroidectomy [11]. Multi-nodular goiter and thyroid cancers are the two main etiologies found in the literature as responsible for acute dyspnea due to tracheal compression [12]. It is recommended that, as much as possible, interventions be performed only on euthyroid patients. Operating while the patient is in a state of active (uncontrolled) hyperthyroidism multiplies the surgical risks due to hormonal instability and changes in the gland. But some situations require a much shorter preparation time. These are extra-thyroid emergency situations (mainly cardiac or ophthalmological), compressive goiter, failures of conventional treatment, or intolerance to synthetic antithyroid drugs [13]. However, to minimize the surgical risks associated with her hyperthyroidism, a preparation was made for our patient using a beta-blocker and corticosteroids [13]. In our observation, our patient presented with clinical and biological hyperthyroidism, but the respiratory emergency forced us to perform an emergency total thyroidectomy [11]. Our patient benefited from a short pre- and intra-operative preparation based on beta-blockers and corticosteroids. 

Several cases of emergency thyroidectomy for compressive goiter have been carried out successfully [14]. The surgery is technically demanding with a greater associated chance of injury to native structures. Total thyroidectomy is the most commonly described surgical procedure in the surgical management of compressive goiters [11,14]. It has been reported that the main complications to be expected in such huge goiters are hemorrhage, recurrent laryngeal nerve injury, and tracheomalacia. 

Post-thyroidectomy tracheomalacia is a serious but rare complication. The incidence of tracheomalacia post thyroidectomy is reported to be between 0.8% and 5.8% [15]. We note in the literature that patients with longstanding goiter, even when benign, are more prone to develop tracheomalacia [16]. 

There has been no consensus on optimal treatment of tracheomalacia caused by external compression in adults. Treatment is unnecessary in most cases. However, if a patient has evidence of severe tracheomalacia, appropriate treatment of this condition is indicated to prevent catastrophic airway collapse. Treatment options include continuous positive airway pressure, tracheostomy, tracheoplasty, airway stent implantation, tracheal segmentectomy, prolonged endotracheal intubation, and so on. There is no uniform treatment standard, and tracheostomy is currently the most commonly used treatment [17,18]. In our case, our patient presented with tracheomalacia, and for this, we established a prolonged endotracheal intubation associated with corticosteroid therapy for 48 hours. 

In the literature, we have several cases of giant papillary carcinoma leading to acute respiratory failure [19,20], but our observation describes a rare scenario involving hyperthyroidism, which, according to ATA recommendations [4], contraindicates thyroid surgery, the only way to save the patient in this situation. This therapeutic conflict describes a medical coin toss that very few surgeons encounter in their careers. There was therefore a defiance of standard protocols, deviating from the rule of prior euthyroidism [4]. This allowed us to save the patient with the help of a multidisciplinary team. The anesthesiologists performed a brief but effective preparation based on beta-blockers and corticosteroids. The endocrine and ENT surgeons achieved a technical feat by performing a total thyroidectomy on a massive and hypervascularized gland without damage to vital organs.

## Conclusions

The management of a giant papillary thyroid carcinoma causing acute respiratory obstruction associated with thyrotoxicosis presents an exceptional clinical challenge. Although standard protocols require prior hormonal stabilization, the life-threatening emergency of asphyxia necessitates immediate intervention. This exceptional case underscores the importance of rapid and coordinated management in complex medical situations. Collaboration between different medical specialties is essential to determine the best therapeutic approach, even in urgent circumstances. Difficult decisions must be made with consideration of all relevant factors to ensure the best possible outcome for the patient.
